# Leading by example: how to empower supervisors as role models

**DOI:** 10.3389/frma.2025.1533630

**Published:** 2025-04-04

**Authors:** Miriam van Loon, Joeri Tijdink, Natalie Evans, Mariëtte Van Den Hoven

**Affiliations:** Department of Ethics, Law and Humanities, Amsterdam University Medical Center, Amsterdam, Netherlands

**Keywords:** role model, supervisor, responsible conduct of research, empowerment, critical autonomy

## Abstract

Supervisors are considered to play a pivotal role in stimulating responsible conduct of research (RCR). Their position as supervisors of PhD candidates offers the opportunity to be good *role models* and show young researchers how to conduct research properly. In this contribution, we delineate what it means to “lead by example.” We inquire how the concept of role modeling is currently applied in the context of supervision in general, and in RCR specifically, and present the perspective of empowerment as a fruitful approach to help determine what role modeling should focus on when aiming to foster a positive research culture.

## 1 Introduction

Supervisors are considered to play a pivotal role in stimulating responsible conduct of research [RCR; All European Academies (ALLEA), 2023; Lerouge and Hol, 2020]. Haven et al. (2022) described how supervision can both positively and negatively impact the personal and professional development of a PhD candidate. Poor supervision can jeopardize a good working relationship or even stimulate misconduct, while good supervision can integrate young researchers into responsible academic practices and positively boost their academic career and personal development (Anderson et al., 2007; Pizzolato and Dierickx, 2023b). Note that their position as supervisors makes them *de facto* role models, and in the literature, this role is frequently mentioned (Haven et al., 2022; Löfström, 2024; Pizzolato and Dierickx, 2023b). This is illustrated by the following quote: “… besides instructing directly about good research practices and boundaries about what can be done or not, supervisors are exemplars for their doctoral candidates” (Pizzolato and Dierickx, 2023a, p. 428). This role of the supervisor as an exemplar is debated in the literature, both in the context of supervision and mentoring. In this study, we used these words interchangeably. Despite the frequent emphasis on the relevance of role modeling, it is not always clear what this role should entail and how to prepare supervisors for it.

Given the widely acknowledged importance of the supervisor's role in doctorate education, one might believe that supervisors are collectively trained by their institutions. However, the acceptance of such training remains slow within the academic community. Considering that RCR training for supervisors is only now receiving the attention it deserves, this is a good moment to outline appropriate perspectives on how to train supervisors. Therefore, in this contribution, we delineate what it means to “lead by example.”

We first focus on the *concept* of role modeling in supervision by examining the use of the concept in general and, more specifically, in the field of RCR. Next, we suggest that the perspective of empowerment is useful for delineating how role modeling can be shaped in such a way that it helps to foster a positive research culture in practice.

## 2 Role modeling in supervision

In the literature, the concept of role modeling in supervision has primarily been discussed in the context of formal education, with a focus on the responsibilities of *teachers* in shaping *student behavior* (although not yet in relation to PhD candidates). Nejati and Shafaei (2018) stated the following: “…the substantial impact that supervisors can have on students through sharing knowledge and instilling ethical values cannot be ignored” (Nejati and Shafaei, 2018, p. 76). Trevino et al. (2000) applied the concept of *ethical leadership* in academic contexts (Trevino et al., 2000). Alaoui et al. (2024) hypothesized that professors taking on an *ethical* leadership role can “reduce the adoption of deviant behaviors by students” (Alaoui et al., 2024, p. 144). The focus on ethical leadership, as a core interpretation of role modeling, puts emphasis on both the *moral manager* and the *moral person* in a leadership role: “…ethical leaders set an example by adopting normatively appropriate behavior, such as honesty, fairness, loyalty, and attention to subordinates. Ethical leaders communicate ethical values to subordinates by reinforcing ethical standards and using rewards and sanctions regarding the adoption or non-adoption of ethical behaviors” (Alaoui et al., 2024, p. 145). The idea of supervisors as ethical leaders has been used in various studies (Arain et al., 2017; Nejati and Shafaei, 2018; Zhang et al., 2023).

In the context of RCR, we did not find any examples of the application of the concept of ethical leadership; however, the concept seems to fit into the ongoing debate on the role modeling of supervisors. The role model function of supervision is considered to be about “showing specific virtues and characteristics” (Pizzolato and Dierickx, 2023a, p. 428) to help influence and shape the behaviors and attitudes of students. The identified virtues relevant to supervision are *honesty, respectfulness, and availability* (Lee et al., 2007; Pizzolato and Dierickx, 2023a). Therefore, similar to the concept of ethical leadership, which focuses on moral qualities (ethical values) and moral behavior, role modeling in supervision within RCR also focuses on moral qualities (virtues) and behavior. VIRT2UE, a research integrity training program that takes a virtue ethics perspective, recognizes the importance of role modeling in fostering moral sensitivity in its training method (Evans et al., 2024). Gibson introduces another aspect of role modeling, stating that role models often demonstrate a commitment to the personal growth of supervisees, help them to stay up-to-date in their field, and aim to enhance their skills. According to Gibson, a role model stimulates the professional and personal growth of supervisees, which requires more than just moral qualities alone (Gibson, 2004).

Recently, another aspect of role modeling in the context of RCR has been emphasized: the idea that a “supervisor should *act* as a role model *by practicing responsible research*, such as sharing data and code” (Haven, 2024, p. 3 italics added). In this context, the behavioral aspect is also highlighted by exemplifying what responsible research entails in practice. However, two observations can be made. First, if role modeling should focus on showing good and responsible behavior in research conduct, then virtues beyond those mentioned above—such as transparency, accountability, and impartiality—are also relevant to this purpose. Second, acting responsibly in research practice requires specific skills and knowledge—for example, in data management, preregistration, or authorship guidelines—in addition to moral qualities. Only then mentors “can raise mentees' awareness of responsible conduct in all stages of the research process” (Pizzolato and Dierickx, 2023b, p. 19).^1^ It is considered an explicit responsibility of mentors to “demonstrate RI with their own daily research activities,” an approach that is also more effective in influencing mentees (Pizzolato and Dierickx, 2023b).

In practice, a gap between the ideas of role modeling and reality can be observed. Abdi spoke of a “(dis)connect between theory and practice. Supervisors are not trained, and often not skilled in new research practices, such as ‘open science”' (Abdi, 2022, p. 122). In addition, early career researchers often hesitate to speak up about issues related to responsible conduct of research, as they occupy hierarchical and vulnerable positions (Van Den Hoven et al., 2023b). In turn, some supervisors acknowledge that they are insufficiently skilled in research integrity, as expressed well in the following quote: “what I got in terms of research integrity, I got it from my students” (Pizzolato and Dierickx, 2023a, p. 438). Haven et al. (2023) raised the (fair) question of who is role modeling for whom in open science practices, as PhD candidates are sometimes better trained in these matters (Haven et al., 2023, p. 4).

What we have learned so far is that the concept of role modeling is primarily interpreted in terms of *moral qualities* (values and virtues) and is mostly concerned with *behavior*. However, with regards to RCR, it is complemented by the expectation to act responsibly, which requires *knowledge* and *skills* to meet the standards of responsible research practices. Supervisors might be lagging in this regard as their PhD candidates now receive training on these matters, while in most institutions, there is no such mandate for supervisors. Offering training to supervisors could be the next step to help them become aware of the desired role in supervision. In the next section, we suggest that incorporating a specific perspective on the supervisory role might help shape such training.

## 3 Empowerment in RCR

In this section, we use the concept of empowerment as a lens for understanding role modeling in supervisory relations. One highly influential source in the literature on empowerment is the work of Paolo Freire. In his famous book *Pedagogy of the Oppressed*, the concept ***conscientizaçado (critical awareness)***is used to describe the process of becoming conscious of “mechanisms in practices of oppression” in order to learn to liberate oneself from these practices. In his view, liberation is a praxis, a process of humanization: the action and reflection of men and women upon their world in order to transform it (Freire and Ramos, 1970). His work has been an inspiration in many different disciplines, and we believe that it also provides a useful framework for reflecting on responsible conduct of research.

Debates on research integrity and responsible research conduct have been fueled by cases of misconduct. Misconduct in academia often seems related to the highly competitive and demanding system, focusing on (individual) achievements (Carson et al., 2013; Musselin, 2005; Tijdink et al., 2014). This system is also highly hierarchical; status and position are accompanied by a certain amount of power (Papatsiba and Cohen, 2020). These power structures contribute to a competitive environment. Early career researchers are particularly vulnerable and dependent on their supervisors, and there is a high dropout rate (Kis et al., 2022). To Freire, it was clear that developing a critical awareness was the only way to escape the oppressive system and to prevent the oppressed from turning into new oppressors. Liberating oneself requires developing the capacity to become critically aware of one's situation and to speak up. The liberating process can only be supported if people adopt a dialogical attitude. Freire focused specifically on the changes needed in the educational system; thus, he suggested that a radical new attitude toward learning must be adopted. Following Freire's footsteps, Lawson (2011) defines empowerment as the development of critical autonomy: (it) “includes the ability to think for oneself, the ability to use theory as a guide to action, and, crucially, the ability to evaluate the circumstances of one's life, including the structural forces that surround us” (Lawson, 2011, p. 90).

In academia, the hierarchical structure of the academic system creates power dynamics that influence practices and researchers' ability to speak up. Individuals might not feel safe addressing issues (Hawkins et al., 2014), people may be unwilling to report malpractice or may feel they have limited opportunities to improve practices due to a lack of power. Learning to develop critical autonomy could empower researchers to speak up and to work according to their personal and professional values.

The dialogical attitude that Freire suggested has implications for both people in power and the powerless: dialogue requires motivation to engage in a dialectical relationship and also a genuine interest in what the other has to say. Communication and reflection are thus crucial skills in training for empowerment. The ability to engage in dialogue must be cultivated, representing another way in which individuals' capacities of need to be developed to bring about changes in practice. It is important to note that the needed change—both in developing critical awareness and a dialogical attitude—is recognized as relevant at different levels: individual, group, and systemic (Israel et al., 1994). On a systemic level, RCR can be jeopardized by the pressure to publish and obtain grants, which can lead to “sloppy science” or even scientific misconduct (Tijdink et al., 2014). On a group level, RCR can be influenced by local policies, norms, and rules within the department regarding how to conduct research. On an individual level, personal skills and capacities influence RCR, such as knowing the right methodologies to use or being trained to be a good leader. This recognition of the different levels on which RCR can be stimulated, both positively and negatively, has been acknowledged in recent debates on research culture (Ivy et al., 2024; Martin et al., 2023). We believe that, using the perspective of empowerment can be fruitful in the context of research practices, as this is a setting with hierarchical structures and power dynamics (Rushforth, n.d.). Developing critical awareness (or critical autonomy) and adopting a dialogical approach seem beneficial in supporting a positive research culture.

The concept of empowerment has been used previously to develop a teaching philosophy in the Horizon 2020 INTEGRITY project, operationalizing empowerment through five rules of thumb: “RCR training *(1) needs to build the capacities of researchers; (2) needs to stimulate the critical autonomy of researchers, enabling them to (3) learn to take control of integrity issues they encounter in practices, to which they will be 4) motivated to pro-actively react and 5) dare to speak up if necessary*” (Van Den Hoven and Theunissen, 2021). The importance of empowerment is implicit in other RCR training programs, such as VIRT2UE and Path2Integrity, which also utilize case-based, dialogical pedagogical approaches (Van Den Hoven et al., 2023a).

### 3.1 Role modeling and empowerment

We believe that an empowerment perspective can be fruitful in multiple ways in a discussion on role modeling. First, the ideal characteristics we distilled from the literature require supervisors to develop a critical awareness of the role they (always) play, as well as to become competent in the responsible conduct of research. Second, the empowerment perspective shows that supervisors can have both upward and downward influence on improving responsible research practices. Supervisors also depend on others in the hierarchy, with decisions on, for example, researcher assessment often made at higher levels. At the same time, there is a relationship of dependence between supervisors and PhD candidates. Supervisors' empowerment can thus be directed upward by reflecting on opportunities to improve research practices or by helping to change the system or institutional culture. One could, for example, stimulate good norms and a positive research culture in one's department, promote transparent and respectful collaborations, and speak up against certain institutional structures that hinder PhD candidates from flourishing. Empowerment of supervisors can also be directed downward by showing how research is conducted in a responsible manner, setting good standards, and helping PhD candidates become skilled researchers (Van Loon and van den Hoven, 2023). Finally, the empowerment perspective recognizes power dynamics and the need to make reforms by means of stimulating critical autonomy and a dialogical attitude. Stimulating a positive research culture can only be achieved if people work in more open and transparent ways, thereby building trust in and within science.

What does this imply for the idea that supervisors need to lead by example? To illustrate the relevance of critical awareness, we offer two examples. First, if supervisors lead by example in RCR by, for instance, preregistering studies but fail to do so in an open and critical manner with their PhD candidates, then they are simply imposing new research standards on their team. Second, if supervisors are excellent researchers but lack RCR skills and are not critically aware of power dynamics in their communication with junior researchers, then it will be challenging for PhD candidates to learn to speak up and to address common practices. An empowering perspective on their role as mentors could therefore encourage a shift in their supervisory style, potentially transforming it into a mutual learning experience with their PhD candidates. As a consequence, more traditional ways of conceiving role models, such as the “master–apprentice relationship,” which is still quite popular, could move to the background, while new ways of interpreting and stimulating certain types of role models might come to the forefront.

### 3.2 Empowerment and training

If role modeling requires specific skills, then these can be developed through training (Haven et al., 2022, 2023; Van Loon and van den Hoven, 2023). Therefore, it is necessary to design and implement more training programs on responsible supervision, as few are currently available, accessible, or scalable for large groups of researchers. In addition, existing programs do not (always) account for cultural differences. Our discussion on expectations regarding role modeling, if embraced, can help in the design and implementation of such training programs. We believe that, ideally, these programs should focus on developing capacities in dialogical communication, fostering critical autonomy, and learning to adhere to (recently changed) RCR standards. In addition, training can help supervisors reflect on the moral qualities they wish to show in their role as researchers and supervisors. One way to address these aspects in a training program is by distinguishing between hard skills and soft skills.

Traditionally, hard skills refer to the technical skills and knowledge that build expertise. In the context of research, it would entail knowledge and skills that are required to conduct research well (Lamri and Lubart, 2023). We interpret hard skills in the context of RCR as the skills and knowledge required to understand the topics and debates relevant to the responsible conduct of research. This approach also refers to *hands-on learning*, such as how to write a Data Management Plan or whom to turn to within one's organization for support. Soft skills usually refer to “interpersonal, human, people, or behavioral skills, and place emphasis on personal behavior and managing relationships between people. Soft skills are primarily affective or behavioral in nature” (Marin-Zapata et al., 2022). Soft skills in a supervisor training program on RCR could address supervisors as academic professionals with the responsibility to model good and responsible behavior. This behavior includes, for example, helping PhD candidates become independent researchers in academia, fostering interpersonal relationships (e.g., how to give and receive feedback), and developing skills in collaboration, reflection, communication, and leadership. Soft skills could also help supervisors become more aware of power differences and address these differences appropriately in their supervision. These skills can be fostered by teaching specific approaches to mitigate power imbalances in interpersonal relationships and by fostering an understanding of how hierarchical power can be wielded and maintained in relationships (Brage et al., 2016). Both soft skills and hard skills can, in their own way, stimulate critical autonomy in supervisors and help them reflect on how to promote this in their PhD candidates. Ideally, in such training programs, the interaction between supervisors and supervisees is stimulated, for example, via assignments that require them to discuss RCR-related topics together.

### 3.3 Reflection

In this contribution, we introduced an empowerment perspective to further explore the aspects of role modeling in supervision. Role modeling involves behaviors rooted in moral qualities, but in RCR debates, “leading by example” also includes acting in a responsible manner in research practice and being able to pass this on to PhD candidates. Furthermore, embracing the empowerment perspective highlights the need for supervisors to develop the skills to meet higher research standards, cultivate critical autonomy, and adopt a dialogical attitude. Given the advantage that PhD candidates may have in skills and knowledge related to RCR (taking mandatory courses), a promising way forward seems to be exploring whether dialogical learning can foster mutual learning in supervisor relationships. To achieve this, training for supervisors seems like a good next step.

## Data Availability

The original contributions presented in the study are included in the article/supplementary material, further inquiries can be directed to the corresponding author.

## Footnotes

^1^We interpret mentors here as supervisors. Mentors and supervisors do not have the same role, and in various countries, their roles are differently defined. We think that, in this manuscript, mentors have a supervising role, as reflected in the quote.
